# Supplementary material for: Manganese homeostasis supports Stenotrophomonas maltophilia oxidative stress defense and replication in Acanthamoeba castellanii phagosomes
Source: Curr Res Microb Sci. 2026 May 12;10:100604. doi: 10.1016/j.crmicr.2026.100604 (PMC13217887; doi:10.1016/j.crmicr.2026.100604)
Supplement: Supplementary file 1 [file mmc1.pdf]

Table S1. Sm18 significantly up- and down-regulated genes from 0 μM to 8 μM of Mn(II) in minimal MOPS medium with 10 μM Iron. Cutoff: adjusted p-value (padj) < 0.01 and Log_2 fold change (Log_2FC) ≥ 1 or ≤ -1.

Locus id	Gene name	Log_2FC	DE	padj	Gene description
V8P27_004008	*mntP*	3.4	Up	$4.2\ e^{-61}$	Manganese efflux pump
V8P27_003797	V8P27_003797	2.0	Up	$1.4\ e^{-08}$	Copper chaperone SCO1/SenC
V8P27_003805	V8P27_003805	1.8	UP	$1.4\ e^{-08}$	HTH-type transcriptional regulator
V8P27_003804	V8P27_003804	1.7	UP	$6.0\ e^{-03}$	DUF6436 domain-containing protein
V8P27_002360	*mntH*	1.0	Down	$2.0\ e^{-05}$	Mn2+/Fe2+ uptake protein

Table S2. Sm18 significantly up- and down-regulated genes from 0 μM to 8 μM of Mn(II) in minimal MOPS medium without Iron. Cutoff: adjusted p-value (padj) < 0.01 and Log_2 fold change (Log_2FC) ≥ 1 or ≤ -1.

Locus id	Gene name	Log_2FC	DE	padj	Gene description
V8P27_004008	*mntP*	4.0	Up	$2.2\ e^{-131}$	Manganese efflux pump
V8P27_001285	V8P27_001285	2.2	Up	$4.0\ e^{-04}$	TonB-dependent receptor
V8P27_004004	V8P27_004004	1.3	Up	$1.8\ e^{-20}$	Major Facilitator Superfamily transporter
V8P27_004005	V8P27_004005	1.3	Up	$4.8\ e^{-11}$	DcaP outer membrane protein
V8P27_001286	V8P27_001286	1.2	Up	$3.7\ e^{-04}$	MerC domain-containing protein
V8P27_000822	*prpE*	1.0	Up	$1.3\ e^{-09}$	propionate-CoA ligase
V8P27_002357	V8P27_002357	4.6	Down	$8.5e^{-122}$	TonB-dependent receptor
V8P27_002358	V8P27_002358	2.8	Down	$5.3\ e^{-03}$	Thioredoxin-fold protein
V8P27_003990	V8P27_003990	2.0	Down	$1.0\ e^{-05}$	MFP of RND efflux pump
V8P27_001267	V8P27_001267	1.8	Down	$6.5\ e^{-03}$	Cation Diffusion Facilitator transporter
V8P27_002373	V8P27_002373	1.5	Down	$8.4\ e^{-09}$	Small Multidrug Resistance transporter
V8P27_003991	V8P27_003991	1.5	Down	$1.8\ e^{-14}$	RND efflux pump
V8P27_002360	*mntH*	1.5	Down	$8.2\ e^{-15}$	Mn2+/Fe2+ uptake protein
V8P27_003989	V8P27_003989	1.4	Down	$4.0\ e^{-04}$	OMF of RND efflux pump
V8P27_001268	*nfi*	1.3	Down	$3.6\ e^{-08}$	Endonuclease V
V8P27_001249	V8P27_001249	1.3	Down	$7.1\ e^{-07}$	Uncharacterized protein
V8P27_001248	*map*	1.2	Down	$1.7\ e^{-12}$	Type I methionyl aminopeptidase

Table S3. Strains used and constructed in this study.

Strain name	Key strain	Reference/source
DH5α	Standard *Escherichia coli* cloning strain. F− endA1 glnV44 thi-1 recA1 relA1 gyrA96 deoR nupG purB20	Laboratory strain collection
S17.1	Standard *Escherichia coli* conjugative donor strain. recA, thiE1, pro-82, endA, hadR17. RP4-2(Km::Tn7, Tc::Mu1)	(1)
Sm18	*Stenotrophomonas maltophilia* strain Sm18. Environmental isolate from Cuernavaca, Morelos, México.	(2)
Neff	*Acanthamoeba castellanii* strain Neff. Environmental isolate from the USA	ATCC 30010
Sm18Δ*02357*	Deletion mutant of the ORF 02357 from *Stenotrophomonas maltophilia* Strain Sm18	This work
Sm18Δ*02358*	Deletion mutant of the ORF 02358 from *Stenotrophomonas maltophilia* Strain Sm18	This work
Sm18Δ*mntP*	Deletion mutant of the ORF mntP from *Stenotrophomonas maltophilia* Strain Sm18	This work
Sm18Δ*mntR*	Deletion mutant of the ORF mntR from *Stenothophomonas maltophilia* Strain Sm18	This work
Sm18/332	*Stenotrophomonas maltophilia* strain Sm18 transformed with the empty vector pSEVA332	This work
Sm18Δ*mntP*/332	Sm18Δ*mntP* deletion mutant transformed with the empty vector pSEVA332	This work
Sm18Δ*mntP*/332::*mntP*	Sm18Δ*mntP* deletion mutant transformed with the vector pSEVA332::*mntP*	This work
Sm18::mTn7TC1_Pc_mScarlet-I	*Stenotrophomonas maltophilia* strain Sm18 derivative tagged with mScarlet-I expressed from the strong constitutive Pc promoter.	(3)
Sm18Δ*02357*::mTn7TC1_Pc_mScarlet-I	Derivative of the Sm18Δ*02357* deletion mutant, tagged with mScarlet-I and expressed under the strong constitutive Pc promoter	This work
Sm18Δ*02358*::mTn7TC1_Pc_mScarlet-I	Derivative of the Sm18Δ*02358* deletion mutant, tagged with mScarlet-I and expressed under the strong constitutive Pc promoter	This work
Sm18Δ*mntP*::mTn7TC1_Pc_mScarlet-I	Derivative of the Sm18Δ*mntP* deletion mutant, tagged with mScarlet-I and expressed under the strong constitutive Pc promoter	This work
Sm18Δ*mntR*::mTn7TC1_Pc_mScarlet-I	Derivative of the Sm18Δ*mntR* deletion mutant, tagged with mScarlet-I and expressed under the strong constitutive Pc promoter	This work

Sm18VIM*02357*	Interrupted mutant of the ORF *02357* from *Stenotrophomonas maltophilia* Strain Sm18	This work
Sm18VIM*mntH*	Interrupted mutant of the ORF mntH from *Stenotrophomonas maltophilia* Strain Sm18	This work
Sm18VIM*mntP*	Interrupted mutant of the ORF mntP from *Stenotrophomonas maltophilia* Strain Sm18	This work
Sm18VIM*mntH*::mTn7TC1_Pc_mScarlet-I	Derivative of the Sm18VIMmntH interrupted mutant, tagged with mScarlet-I and expressed under the strong constitutive Pc promoter	This work
Sm18VIM*mntH*/332	Sm18VIMmntH interrupted mutant transformed with the empty vector pSEVA332	This work
Sm18VIM*mntH*/332::mntH	Sm18VIMmntH interrupted mutant transformed with the vector pSEVA332::*mntH*	This work
Sm18/327	*Stenotrophomonas maltophilia* strain Sm18 transformed with the empty expression vector pSEVA327	This work
Sm18/327-pr_*02357-02358*	*Stenotrophomonas maltophilia* strain Sm18 transformed with the expression vector pSEVA327-pr_*02357-02358*	This work
Sm18/327-pr_*mntH*	*Stenotrophomonas maltophilia* strain Sm18 transformed with the expression vector pSEVA327-pr_*mntH*	This work
Sm18/327-pr_*mntP*	*Stenotrophomonas maltophilia* strain Sm18 transformed with the expression vector pSEVA327-pr_*mntP*	This work
Sm18/337R	*Stenotrophomonas maltophilia* strain Sm18 transformed with the empty expression vector pSEVA337R	This work
Sm18/337R-pr_*02357-02358*	*Stenotrophomonas maltophilia* strain Sm18 transformed with the expression vector pSEVA337R-pr_*02357-02358*	This work
Sm18/337R-pr_*mntH*	*Stenotrophomonas maltophilia* strain Sm18 transformed with the expression vector pSEVA337R-pr_*mntH*	This work
Sm18/337R-pr_*mntP*	*Stenotrophomonas maltophilia* strain Sm18 transformed with the expression vector pSEVA337R-pr_*mntP*	This work
Sm18∆*mntR*/327	Derivative of the Sm18∆*mntR* deletion mutant transformed with the empty expression vector pSEVA327	This work
Sm18∆*mntR*/327-pr_*02357-02358*	Derivative of the Sm18∆mntR deletion mutant transformed with the expression vector pSEVA327-pr_*02357-02358*	This work

Sm18Δ*mntR*/327-pr_*mntH*	Derivative of the Sm18ΔmntR deletion mutant transformed with the expression vector pSEVA327-pr_*mntH*	This work
Sm18Δ*mntR*/327-pr_*mntP*	Derivative of the Sm18ΔmntR deletion mutant transformed with the expression vector pSEVA327-pr_*mntP*	This work

Table S4: Vectors used and constructed in this study

**Plasmid name**	**Plasmid features**	**Reference/source**
pUC18T_mTn7TC1_Pc_mScarlet-I	pUC18T_mTn7TC1_Pr_mScarlet-I derivative for chromosomal labeling of bacteria with constitutive mScarlet-I expression driven from the strong Pc promoter; 5,893 bp.	(3)
pTNS2	R6K-based plasmid (ApR) encoding the TnsABCD Tn7 transposase expression genes; 9,615 bp.	AddGene #64968
pEX18Tc	Mobilizable plasmid containing OriT, for site-targeted mutagenesis in Gram-negative bacteria; 6349 bp.	(4)
Sm18Δ*02357*_pEX18Tc	pEX18Tc derivative with homologous DNA genetic fragments flanking the ORF *02357* of Sm18; 7424 bp.	This work
Sm18Δ*02358*_pEX18Tc	pEX18Tc derivative with homologous DNA genetic fragments flanking the ORF *02358* of Sm18; 7343 bp.	This work
Sm18Δ*mntP*_pEX18Tc	pEX18Tc derivative with homologous DNA genetic fragments flanking the ORF *mntP* of Sm18; 7375 bp.	This work
Sm18Δ*mntR*_pEX18Tc	pEX18Tc derivative with homologous DNA genetic fragments flanking the ORF *mntR* of Sm18; 7340 bp.	This work
pEX18TcVIM_GFP_PEM7	pEx18Tc derivative with expression of GFP under control of constitutive promoter PEM7 and deletion of the gen SacB; 6031 pb.	Laboratory constructions collection
Sm18VIM*02357*_pEX18TcΔSacB7_PEM7	pEX18TcΔ*SacB7*_PEM7 derivative with homologous fragment of the ORF *02357* of Sm18; 6404 bp.	This work
Sm18VIM*mntH*_pEX18TcΔSacB7_PEM7	pEX18TcΔ*SacB7*_PEM7 derivative with homologous fragment of the ORF *mntH* of Sm18; 6380 bp.	This work
Sm18VIM*mntP*_pEX18TcΔSacB7_PEM7	pEX18TcΔ*SacB7*_PEM7 derivative with homologous fragment of the ORF *mntP* of Sm18; 6360 bp.	This work
pSEVA332	Empty vector (chloramphenicol resistance, ori pBBR1, cargo lacZα-pUC19); 3417 bp.	(5)
pSEVA332::*mntH*	pSEVA332 digested with PacI and SpeI to remplace the lacZα-pUC19 cargo with mntH gene; 4452 bp.	This work

pSEVA332::*mntP*	pSEVA332 digested with PacI and SpeI to remplace the lacZα-pUC19 with *mntP* gene; 4077 bp.	This work
pSEVA327	Empty expression vector (chloramphenicol resistance, ori RK2, cargo GFP); 4408 bp.	(5)
pSEVA327-pr_*02357-02358*	pSEVA327 containing the promoter region of the ORF *02357-02358*; 4796 bp.	This work
pSEVA327-pr_*mntH*	pSEVA327 containing the promoter region of the ORF *mntH;* 4721 bp.	This work
pSEVA327-pr_*mntP*	pSEVA327 containing the promoter region of the ORF *mntP*; 4976 bp.	This work
pSEVA337R	Empty expression vector (chloramphenicol resistance, ori pBBR1, cargo mCherry); 3661 bp.	(5)
pSEVA337R-pr_*02357-02358*	pSEVA337R containing the promoter region of the ORF *02357-02358*; 4049 bp.	This work
pSEVA337R-pr_*mntH*	pSEVA337R containing the promoter region of the ORF *mntH*; 3974 bp.	This work
pSEVA337R-pr_*mntP*	pSEVA337R containing the promoter region of the ORF *mntP*; 4229 bp.	This work

Table S5: Primers designed in this study for constructing vectors

Primer name	Primer sequence (5' to 3')	Restriction sites	Reference/source
Sm18_D02361_Fragment1.F	cgttgtaaaacgacggccagtgccatgtttggccagagc	None	This work
Sm18_D02361_Fragment1.R	tcgcagtgcttcacatgaaggcgtcgtcct	None	This work
Sm18_D02361_Fragment2.F	gacgccttcatgtgaagcactgcgagcgc	None	This work
Sm18_D02361_Fragment2.R	tctagagtcgacctgcaggcatgcatttcgcgcgaccc	None	This work
Sm18_D02362_Fragment1.F	tgtaaaacgacggccagtgccaaaaccaacctcggctgg	None	This work
Sm18_D02362_Fragment1.R	cgcgaccctaccaacagcgctcgcagt	None	This work
Sm18_D02362_Fragment2.F	gagcgctgttggtagggtcgcgcgaa	None	This work
Sm18_D02362_Fragment2.R	agagtcgacctgcaggcatgcatcgctgaaaagcacccccctt	None	This work
Sm18DmntP_PCR1.FOR	cgttgtaaaacgacggccagtgccacgcttacgacctcaacgtct	None	This work

Sm18DmntP_PCR1.REV	cgcggaagcgttaaatgggggacatggacagc	None	This work
Sm18DmntP_PCR2.FOR	atgtcccccatttaacgcttccgcgattacgt	None	This work
Sm18DmntP_PCR2.REV	ggaaacagctatgaccatgattacgcgatgttctcgatcattctgccg	None	This work
Sm18_DmntR_Fragment1.F	tgtaaaacgacggccagtgccaaacagcgctcgctgcgaccga	None	This work
Sm18_DmntR_Fragment1.R	gcgcaggcgtgtagcggtggcgccgg	None	This work
Sm18_DmntR_Fragment2.F	cgccaccgctacacgcctgcgcctg	None	This work
Sm18_DmntR_Fragment2.R	agagtcgacctgcaggcatgcaatcacttcggccaggtcgcaggcgatgatcgc	None	This work
Sm18_VIM_02361.F	aaaaaGAATTCcacacccctcttccatcgg	EcoRI	This work
Sm18_VIM_02361.R	aaaAAGCTTcttgaccacttcgatgcgg	HindIII	This work
Sm18-VIM-mntH.F	aaaaAAGCTTctacatgatctcggtcggct	HindIII	This work
Sm18-VIM-mntH.R	aaaaGGATCCgaagatcaccatcagcagcg	BamHI	This work
Sm18-VIM-mntP.F1	aaaaAAGCTTccccatttcgatcctcctga	HindIII	This work
Sm18-VIM-mntP.R1	aaaaGGATCCcgccgatatgcacatccatg	BamHI	This work
Sm18_mntHcompl.f	aaaaTTAATTAAtagtcctccaccagctccatc	PacI	This work
Sm18_mntHcompl.R	aaaaACTAGTaaggcatccacgcatggcg	SpeI	This work
Sm18_mntPcompl.F1	aaaaTTAATTAAgcttacgacctcaacgtc	PacI	This work
Sm18_mntPcompl.R	aaaaACTAGTatcgcgtgctgaccaagcc	SpeI	This work
Sm18_pr_02357.F	aaaaaGAATTCtaccgcaaccatggagcc	EcoRI	This work
Sm18_pr_02357.R	aaaAAGCTTctgactttcacctgcacgc	HindIII	This work
Sm18-pr-mntH-p3.f	atatGGATCCtagtcctccaccagctccatc	BamHI	This work
Sm18-pr-mntH-p3.R	atatAAGCTTaaccaccagtgacccttgtcg	HindIII	This work
Sm18-pr-mntP.F1	atataGAATTCgcttacgacctcaacgtc	EcoRI	This work
Sm18-pr-mntP.R1	atatGGATCCgatcaggaggatcgaaatg	BamHI	This work

TABLE S6: Primers used and synthesized in this study to verify constructions or mutants.

Primer name	Primer sequence (5' to 3')	Mutant/Constructions	Reference/source

F24	cgccagggttttcccagtcacgac	Forward primer for confirming constructs or merodiploids strains in plasmids pEXTc18 and constructs in pEXTc18ΔSacB7_PEM7	(6)
R24	agcggataacaatttcacacagga	Reverse primer for confirming constructs in plasmids pEXTc18, insertional mutations in pEXTc18ΔSacB7_PEM7 and Forward primer for confirming transcriptional fusions in pSEVA327 and pSEVA 337R	(6)
pBBR1_VER.F	cggccatcgtccacatatcc	Forward primer for confirming constructs in plasmid pSEVA332	This work
Sm18_D02361_VER.F	atgttctgatcccgctttcg	Forward primer for confirming deletion mutant Sm18Δ02357	This work
Sm18_D02361_VER.R	tgacgtacaccacttcggtatc	Reverse primer for confirming deletion mutant Sm18Δ02357	This work
Sm18_D02362_VER.F	gctcgtggtacgtcaacctg	Forward primer for confirming deletion mutant Sm18Δ02358	This work
Sm18_D02362_VER.R	gtcgagctgatctcggacct	Reverse primer for confirming deletion mutant Sm18Δ02358	This work
Sm18_DmntP_VER.F	gctatacaaaccagccctgc	Forward primer for confirming deletion mutant Sm18ΔmntP	This work
Sm18_DmntP_VER.R	tggatcaggcgttggagaaa	Reverse primer for confirming deletion mutant Sm18ΔmntP	This work
Sm18_DmntR_VER.F	agcctggatgagagcgagag	Forward primer for confirming deletion mutant Sm18ΔmntR	This work
Sm18_DmntR_VER.R	ttcatggggccgaatatagc	Reverse primer for confirming deletion mutant Sm18ΔmntR	This work
Sm18_M02362_VER.R	tccaggctgacgtacaccac	Reverse primer for confirming merodiploids Sm18::pEX18Tc-02358	This work
Sm18_MmntR_VER.R	agaccgtggagcgattcct	Reverse primer for confirming merodiploids Sm18::pEX18Tc-mntR	This work

Steno_glmS_down.F (Smal_glmS_down_1549F)	gacatgccggtggtggtgatcg	Forward primer for confirming insertion of pUC18T_mTn7TC1_Pc_mScarlet-I in Sm18 Strains	(3)
pTn7.R	cacagcataactggactgatttc	Reverse primer for confirming insertion of pUC18T_mTn7TC1_Pc_mScarlet-I in Sm18 Strains	(3)

References

1. Simon R, Priefer U, Pühler A. 1983. A Broad Host Range Mobilization System for In Vivo Genetic Engineering: Transposon Mutagenesis in Gram Negative Bacteria. Nat Biotechnol 1:784–791.

2. Ochoa-Sánchez LE, Vinuesa P. 2017. Evolutionary Genetic Analysis Uncovers Multiple Species with Distinct Habitat Preferences and Antibiotic Resistance Phenotypes in the Stenotrophomonas maltophilia Complex. Front Microbiol 8:1548.

3. Rivera J, Valerdi-Negreros JC, Vázquez-Enciso DM, Argueta-Zepeda F-S, Vinuesa P. 2024. Phylogenomic, structural, and cell biological analyses reveal that *Stenotrophomonas maltophilia* replicates in acidified Rab7A-positive vacuoles of *Acanthamoeba castellanii*. Microbiol Spectr 12:e02988-23.

4. Hoang TT, Karkhoff-Schweizer RR, Kutchma AJ, Schweizer HP. 1998. A broad-host-range Flp-FRT recombination system for site-specific excision of chromosomally-located DNA sequences: application for isolation of unmarked Pseudomonas aeruginosa mutants. Gene 212:77–86.

5. Martínez-García E, Fraile S, Algar E, Aparicio T, Velázquez E, Calles B, Tas H, Blázquez B, Martín B, Prieto C, Sánchez-Sampedro L, Nørholm MHH, Volke DC, Wirth NT, Dvořák P, Alejaldre L, Grozinger L, Crowther M, Goñi-Moreno A, Nikel PI, Nogales J, de Lorenzo V. 2023. SEVA 4.0: an update of the Standard European Vector Architecture database for advanced analysis and programming of bacterial phenotypes. Nucleic Acids Research 51:D1558–D1567.

6. Silva-Rocha R, Martínez-García E, Calles B, Chavarría M, Arce-Rodríguez A, De Las Heras A, Páez-Espino AD, Durante-Rodríguez G, Kim J, Nikel PI, Platero R, De Lorenzo V. 2013. The Standard European Vector Architecture (SEVA): a coherent platform for the analysis and deployment of complex prokaryotic phenotypes. Nucleic Acids Research 41:D666–D675.
